# Comparison of circulating fibrocytes from non-asthmatic patients with seasonal allergic rhinitis between in and out of pollen season samples

**DOI:** 10.1186/s13223-022-00663-5

**Published:** 2022-03-16

**Authors:** Marie-Ève Côté, Marie-Ève Boulay, Sophie Plante, Andréanne Côté, Jamila Chakir, Louis-Philippe Boulet

**Affiliations:** Institut Universitaire de Cardiologie et de Pneumologie de Québec-Université Laval, 2725, Chemin Ste-Foy, Québec, QC G1V 4G5 Canada

**Keywords:** Allergic rhinitis, Airway remodeling, Seasonal allergen exposure, Fibrocyte

## Abstract

**Background:**

Allergic rhinitis is a risk factor for asthma development. In asthma, fibroblast progenitors, fibrocytes, are increased in the blood and bronchial mucosa following allergen exposure. These cells may play a role in lower airways remodeling as observed in non-asthmatic subjects with allergic rhinitis.

**Objective:**

To determine the influence of seasonal allergen exposure on blood circulating fibrocytes in allergic rhinitic subjects without asthma.

**Methods:**

Non-asthmatic subjects with seasonal allergic rhinitis had blood sampling at baseline and at the peak of rhinitis symptoms. Cells were stained for fibrocyte markers (CD34, CD45, CXCR4, collagen I) and analyzed by flow cytometry.

**Results:**

Data from 26 subjects (11M:15F) aged 29 ± 8 years were analysed. Compared to baseline, there was a significant decrease in blood fibrocytes during the pollen season in subjects sensitized to trees [median (25–75 percentile), 9.3 (6.4–20.7)% vs 7.0 (4.2–10.1)%, *P* = 0.007] and a significant increase in subjects sensitized to grass [12.7 (9.9–23.1)% vs 64.0 (57.6–73.6)%, *P* < 0.001] and ragweed [8.0 (7.4–10.8)% vs 48.2 (43.5–52.6)%, *P* < 0.001]. A significant decrease in CXCR4 mean fluorescence was also observed between the two visits [1814 (1261–2235) vs 1352 (814–1796) (arbitrary units), *P* = 0.02].

**Conclusions and clinical relevance:**

These results contribute to document dynamic variations in blood fibrocytes’ activation and migration into the airways following natural exposure to allergens. These findings may help identify one of the potential factors involved in the development of asthma in allergic rhinitic subjects.

## Introduction

Allergic rhinitis is a global health problem, affecting 10–40% of the population, and a major risk factor in the development of asthma [1]. Indeed, patients with allergic rhinitis have a three-fold increased risk of developing asthma and over 80% of asthmatic patients have allergic asthma [2]. Patients with allergic rhinitis may show asthmatic features such as airway hyperresponsiveness and lower airway inflammation and remodeling, but to a lesser extent than in asthma [3, 4]. Although the link between allergic rhinitis and asthma has been extensively studied, we still do not know why some rhinitic patients develop asthma and others do not.

Patients with seasonal allergic rhinitis show increased lower airway inflammation during natural pollen exposure compared to out of season [5, 6]. Allergic patients without asthma also show structural changes in the bronchial mucosa such as sub-epithelial collagen and fibronectin deposition, but to a lesser extent than in asthma [7]. This bronchial remodeling process involves the production of extracellular matrix (ECM) components by fibroblasts and myofibroblasts which accumulate in the bronchial wall during the process of fibrosis [8].

Fibrocytes are the precursor cells of fibroblasts and myofibroblasts. These bone marrow progenitor cells circulate in the peripheral blood to the injured tissue site [9]. The number of fibrocytes increases in the peripheral blood and the bronchial mucosa according to asthma severity and in asthmatics following allergen exposure [9, 10]. Indeed, in allergic asthma, allergen exposure can trigger the release of fibrocytes in the peripheral blood after laboratory bronchial allergen challenge [11, 12]. Furthermore, their accumulation correlates with the decline in lung function and with the progressive development of a component of a fixed airway obstruction [13].

The aim of this study was therefore to determine the effect of a natural seasonal allergen exposure on the profile of circulating fibrocytes isolated from blood of allergic rhinitic patients without asthma.

## Methods

### Subjects

Non-smoking subjects aged 18–55 years with a history of seasonal allergic rhinitis were recruited. All had a positive response to skin prick tests for at least one type of seasonal aeroallergens (trees: ash, poplar, birch, elm, maple, oak; grasses: grasses, timothy, perennial ryegrass; and/or ragweed) and a methacholine PC_20_, the provocative concentration giving a 20% fall in forced expiratory volume in one second (FEV_1_), > 16 mg/ml at the baseline visit. No subject had a history of asthma or used asthma medication in the past. None had a recent (< 1 month) upper or lower respiratory tract infection, another active chronic inflammatory disease, or had received allergen specific immunotherapy injections during the study. All subjects gave their written informed consent.

### Study design

The study was performed at the research center of the Institut Universitaire de Cardiologie et de Pneumologie de Québec-Université Laval (IUCPQ-UL) in Quebec city and included three visits: two visits were performed out of the pollen season (November–March) and one visit was done during the pollen season (April–October) at the peak of rhinitis symptoms before taking any medication for allergies. At inclusion in the study (out of the pollen season), medical history—including demographic information, medication review and physical examination- was recorded, rhinitis control was assessed, rhinitis severity was determined, and skin prick tests, spirometry and methacholine bronchoprovocation were done. Seven to 10 days following this screening visit, subjects came back for blood sampling, nasal inspiratory flow measurement and sputum induction. The same tests were performed during the pollen season, when the participants felt that their allergy symptoms were at worst according to their knowledge of their own history of allergic rhinitis, and when they felt the need to take medication. The main symptoms of allergic rhinitis were explained to the patients prior to their in-season visit to ensure that they recognized the peak of their symptoms. As some subjects were sensitized to more than one seasonal allergen, those who had completed the pollen season visit during the grasses or ragweed pollen season could have experienced symptoms of allergies earlier if they were sensitized to tree pollens, although not strong enough to consider they were at their peak of symptoms. Thus, subjects who completed the visit during the grasses or ragweed pollen season had a longer exposure time than those who completed their visit during the tree pollen season. Pollen forecast was obtained from Aerobiology Research Laboratories, through MeteoMedia’s website (http://www.meteomedia.com). Ethical clearance was obtained from the ethics committee of the IUCPQ-UL.

### Rhinitis control scoring system (RCSS)

The rhinitis control scoring system (RCSS), a brief, validated, subject-completed tool including 5 items (sneezing, rhinorrhea, nasal obstruction, nasal pruritus, and conjunctivitis) was used to assess rhinitis control [14]. Each symptom is rated on a 5-point scale depending on its intensity (none—10%, mild—8%, moderate—6%, severe—4%, very severe—2%) and its frequency (never—10%, rarely—8%, occasionally—6%, frequently—4%, very frequently—2%), which are assessed separately. The sum of the intensity score and the frequency score gives the global score, which ranges from 20 to 100, 100 being a complete control. Controlled rhinitis was defined as a ≥ 80% global score.

### Rhinitis severity

Rhinitis was defined according to the Allergic Rhinitis and its Impact on Asthma (ARIA) guidelines [1]. For mild allergic rhinitis, none of the following items were present and for moderate/severe rhinitis, one item or more were present: (1) sleep disturbance, (2) impairment of daily activities, leisure and/or sport, (3) impairment of school or work and (4) troublesome symptoms.

### Skin prick tests

If not done in the 2 years preceding study visit, atopy was determined from skin prick tests, performed with 25 common aeroallergens. Normal saline and histamine were used as negative and positive controls, respectively. Skin wheal diameter was recorded after 10 min and a positive response was defined as a skin wheal diameter ≥ 3 mm.

### Nasal peak inspiratory flow (NPIF)

NPIF was measured with a nasal peak flow meter (In-Check, Clement-Clarke, UK), using the method described by Youlten [15]. The best of 3 reproducible measurements (≤ 10% difference) was recorded.

### Spirometry

Baseline FEV_1_ and forced vital capacity (FVC) were measured according to the American Thoracic Society (ATS) criteria [16]. FEV_1_ was defined as the best of 3 reproducible values (± 5% and 150 ml) and the predicted values were obtained from the European Respiratory Society Global Lung Function Initiative (GLI-2012) [17, 18].

### Methacholine bronchoprovocation

Airway responsiveness to methacholine was measured using the “classical” tidal volume method described by Juniper et al. [19]. Briefly, following a 2-min inhalation of 0.9% saline, increasing concentrations of methacholine were inhaled for 2 min via a Wright nebulizer (Roxon Meditech, Montreal, QC, Can) delivering 0.13 ml/min. FEV_1_ was measured at 30 and 90 s following inhalation or until FEV_1_ had increased. The test was stopped when a ≥ 20% fall in FEV_1_ from the lowest post-saline value was obtained or when the last methacholine dose was given (64 mg/ml). The response was expressed as the PC_20_ methacholine obtained from the log dose–response curve.

### Sputum induction and processing

Sputum was induced by inhalation of hypertonic saline and processed using the method described by Pin et al. [20] and modified by Pizzichini et al. [21]. Briefly, mucus plugs were selected from saliva, weighed, and treated with dithiothreitol (DTT). Following filtration, total cell count and viability were determined. Two slides were prepared and stained with Diff-Quik for differential cell count.

### Blood sampling

A 200 ml peripheral blood sampling was taken for fibrocyte analysis, complete blood count and serum IgE levels. EDTA plasma and serum from clotted blood were also obtained for mediators’ measurements after a centrifugation at 2000*g* for 10 min at 4 °C.

### Fibrocytes isolation and characterization

Blood (180 ml) was layered on Lymphocyte Separation Medium (Corning, Tewksbury, MA, USA) with a centrifugation for 17 min at 350 g, and the peripheral blood mononuclear cells (PBMC) in the interface were isolated. PBMC were washed using Hank’s Balanced Salt Solution (HBSS). Cells (40,000–50,000 cells) were resuspended in Phosphate Buffered Saline (PBS) with 2 mM EDTA and 0.25% Bovine Serum Albumin (BSA), stained with anti-CD3 microbeads (Miltenyl Biotech, Auburn, CA, USA), and added to a LD column for a magnetic separation (Miltenyl Biotech) to eliminate CD3 positive cells. Cells (50,000—100,000 cells) were resuspended in HBSS containing 2% Fetal Bovine Serum inactivated (FBSi) and were incubated at 4 °C (1) for 30 min with anti-collagen type I (EMD Millipore, Temecula, CA, USA), (2) for 20 min with APC goat anti-mouse Ig (BD Pharmingen, San Jose, CA, USA), and (3) for 30 min with FITC mouse anti-human CD34, APC-Cy7™ mouse anti-human CD45 (BD Pharmingen) and Brillant Violet 42™ anti-human CD184 (CXCR4) (BioLegend, San Diego, CA, USA). A negative control, single antibody controls and an aliquot with a combination of all antibodies were added. Cells were analyzed by flow cytometry. Data were acquired with the BD LSRFortessa cell analyser, using FACSDiva software (BD Biosciences, San Jose, CA): debris were first eliminated from the gated CD3- population. Then, the number of CD34^+^ cells expressing CD45 and collagen I was assessed and the expression of CXCR4 on the surface of these cells was obtained by measuring mean fluorescence. A minimum of 20,000 cells was analyzed per condition and 50,000 cells for the aliquot with the mixed antibodies. The gates for the CD34^+^CD45^+^Col1^+^ cells were established according to the negative and single antibody controls.

### Mediator measurements

Stem cell factor [SCF; minimum detectable dose (MDD) = 9.0 pg/ml], matrix metalloproteinase 9 (MMP-9; MDD = 0.156 ng/ml), tissue inhibitor of metalloproteinase 1 (TIMP-1; MDD = 0.08 ng/ml) and CXCL12 (MDD = 47 pg/ml) (R&D Systems, Minneapolis, MN, USA) were measured in serum or plasma by enzyme-linked immunosorbent (ELISA) assay according to the manufacturer’s instructions. Analyses were done on Synergy H1 Multi-Mode Reader, using Gen5 Microplate Reader and Imager Software (BioTek Instruments Inc., VT, USA). Data were only accepted if the coefficient of variation was ≤ 20%.

### Statistical analyses

Continuous and nominal variables were expressed using mean ± SD and number (%), respectively. Data were analyzed using a two-way mixed model. Two experimental factors were defined; one associated to the comparison between groups and one associated to the comparison between visits, factors fixed, with interaction terms between the fixed factors. When effect that specifies heterogeneity in the covariance structure was significant (heteroscedasticity) compared to the same variance between groups, the statistical analyses were performed using separate residual variance per group. The Satterthwaite’s degree of freedom statement was added for unequal variance structures. For variables for which normality assumption was not fulfilled, analyses were performed on an appropriate transformation (log, square root). The multivariate normality assumptions were verified with the Shapiro–Wilk tests after a Cholesky factorization on residuals. The results were considered significant with *P* values ≤ 0.05. All analyses were conducted using the statistical package SAS, version 9.4 (SAS Institute Inc., Cary, NC, USA) and R [R Core Team (2016), Foundation for Statistical Computing, Vienna, Austria].

## Results

### Subjects’ recruitment

**Fig. 1:**
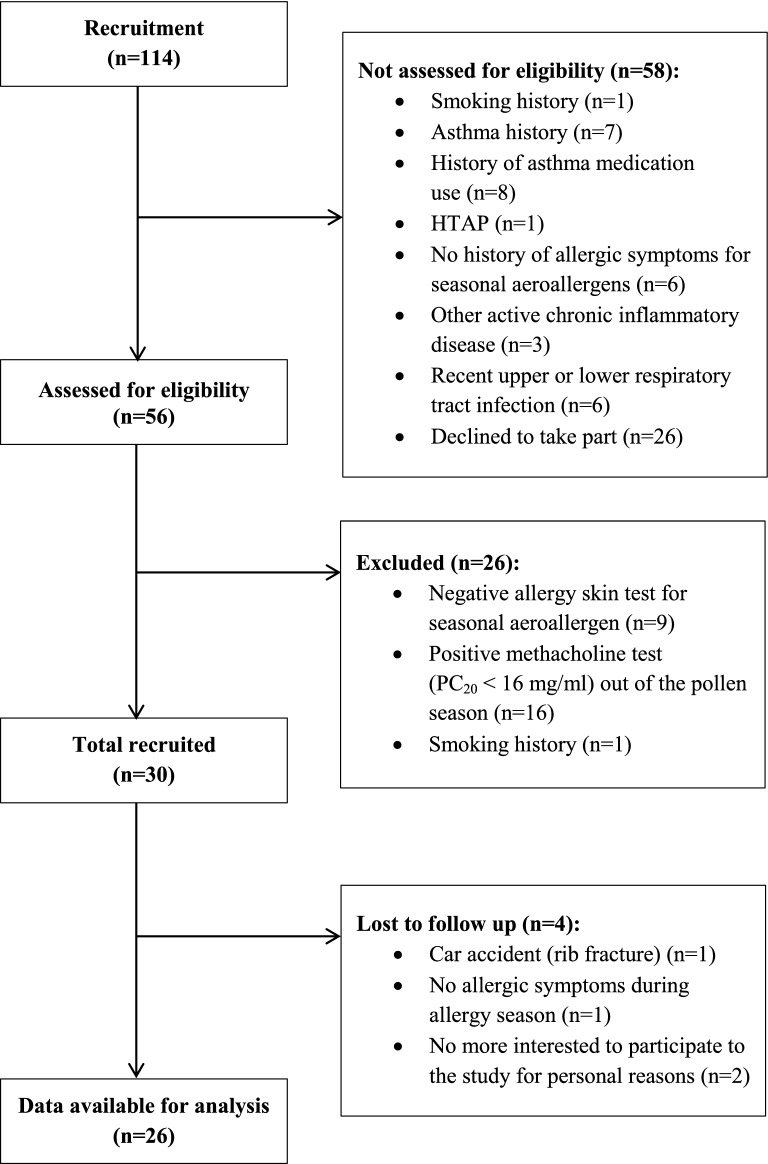
Recruitment flow chart

Among the 56 subjects recruited, 30 were eligible (Fig. 1). Four subjects did not complete the study; hence, 26 subjects were included in the final analyses.

### Characteristics of subjects

Characteristics of subjects at baseline and during the pollen season are presented in Table 1. Among the 26 subjects, mean age was 29 years, 11 were males, 23 had a moderate to severe allergic rhinitis and all had normal lung function. As the peak of symptoms was associated with three main allergen types, participants were therefore separated into three groups for further analyses (1) tree pollen: subjects sensitized to tree pollen and being evaluated during the tree allergy season, (2) grass pollen: subjects sensitized to grass pollen and being evaluated during the grass allergy season, and (3) ragweed pollen: subjects sensitized to ragweed pollen and being evaluated during the ragweed allergy season. These groups were not pre-established. Three subjects of the grass pollen season group were also sensitized to tree pollen and 6 subjects of the ragweed pollen season group were also sensitized to tree and/or grass pollen.

**Table 1:** Subjects' clinical and inflammatory characteristics out and during the pollen season

	Out of the pollen season	During the pollen season	*P* value
RCSS Score (%)	85 ± 14	59 ± 13	< 0.001
PC_20_ (mg/mL) [min–max]	[19.59–> 64]	[5.23–> 64]	
FEV_1_ (% predicted)	100 ± 13	99 ± 13	NS
FVC (% predicted)	107 ± 14	105 ± 13	NS
FEV_1_/FVC (%)	0.79 ± 0.05	0.79 ± 0.05	NS
Nasal peak flow (L/min)	134 ± 45	126 ± 53	NS
Serum IgE (UI/ml)	111 ± 87	118 ± 90	NS
Blood eosinophils (× 10^9^ cells/L)	0.15 ± 0.11	0.18 ± 0.11	0.02
Sputum eosinophils (%) (n = 8)	1.47 ± 2.44	2.06 ± 1.71	NS
CXCR4 circulating fibrocytes [mean fluorescence, median (25–75 Percentile)]			
All subjects (n = 26)	1814 [1261–2235]	1352 [814–1796]	0.02
Tree pollen (n = 12)^a^	1814 [1289–1995]	1762 [1282–1963]	NS
Grass pollen (n = 6)^a^	1798 [1633–2074]	744 [668–925]	< 0.001
Ragweed pollen (n = 8)^a^	2022 [1170–2572]	1394 [629–1507]	0.01
CXCL12 (plasma) (pg/mL)	2650 ± 485	2643 ± 475	NS
SCF (serum) (pg/mL)	828 ± 146	826 ± 151	NS
MMP-9/TIMP-1 (serum)	2.08 ± 0.91	2.21 ± 1.12	NS

Data are expressed as mean ± SD, unless stated otherwise

*CXCL12* cxc-motif chemokine ligand 12; *FEV*_*1*_ forced expiratory volume in one second; *FVC* forced vital capacity; *IgE* immunoglobulin E; *MMP-9* matrix metalloproteinase 9; *NS* not significant; *PC*_*20*_ provocative concentration giving a 20% fall in forced expiratory volume in one second; *RCSS* rhinitis control scoring system; *SCF* stem cell factor; *TIMP-1* tissue inhibitor of metalloproteinase 1

^a^Subjects sensitized to this pollen type and being evaluated during its allergy season

At the peak of symptoms, compared to baseline, subjects showed a significantly lower RCSS score (worse condition) and a statistically significant increase in blood eosinophils, although total IgE levels were similar between the two visits (Table 1). No differences in nasal peak flow values or lung function were observed between baseline and peak of symptoms. Although there was no difference in methacholine PC_20_ between visits, a significant decrease in PC_20_ was observed in 6 subjects at peak of symptoms with a difference of at least one doubling concentration of methacholine. Among these subjects, three had a PC_20_ > 16 mg/ml out of the pollen season and a PC_20_ < 16 mg/ml during the pollen season. No difference in sputum eosinophils percentage was observed between visits.

### Pollen counts

**Fig. 2:**
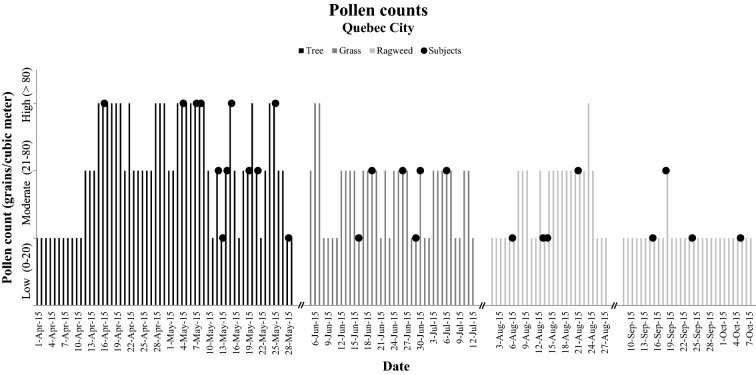
Pollen counts during the pollen season. The dots represent the visit of each subject. The columns are identified according to the pollen period

In Quebec City, tree pollen is the first type of pollen to appear during the spring. Therefore, it is the first exposure of the year to seasonal allergens, followed by grasses and ragweed. The total amount of pollen grains tended to be higher in the tree pollen season than during the grass and ragweed pollen seasons. The amount of tree pollen grains was high (> 80 grains/m^3^) for 6 of 12 subjects during the pollen season visit, and was always moderate (21–80 grains/m^3^) or low (0–20 grains/m^3^) for the subjects exposed to grasses or ragweed (Fig. 2).

### Circulating fibrocytes characterization

**Fig. 3:**
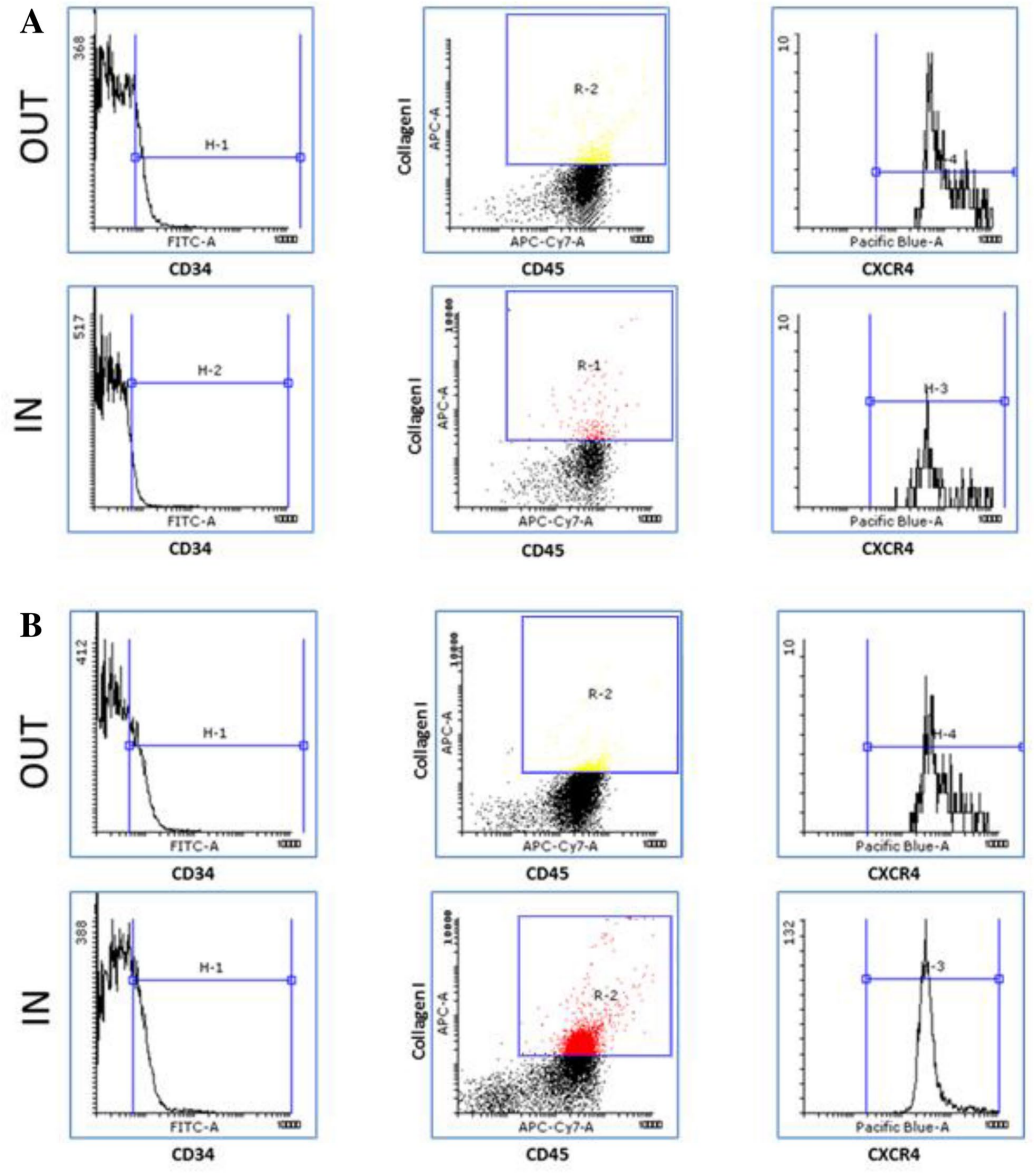
Representative flow cytometry analyses of circulating fibrocytes showing the gating strategy, out vs in **A** tree pollen season and **B** grass or ragweed pollen season. Progenitor cells were selected based on the expression of CD34 (CD34 plots). Fibrocytes were selected afterward with gating of the CD45^+^ collagen I^+^ population (CD45 plots). Finally, the expression of the chemokine receptor CXCR4^+^ was measured (CXCR4 plots)

**Fig. 4:**
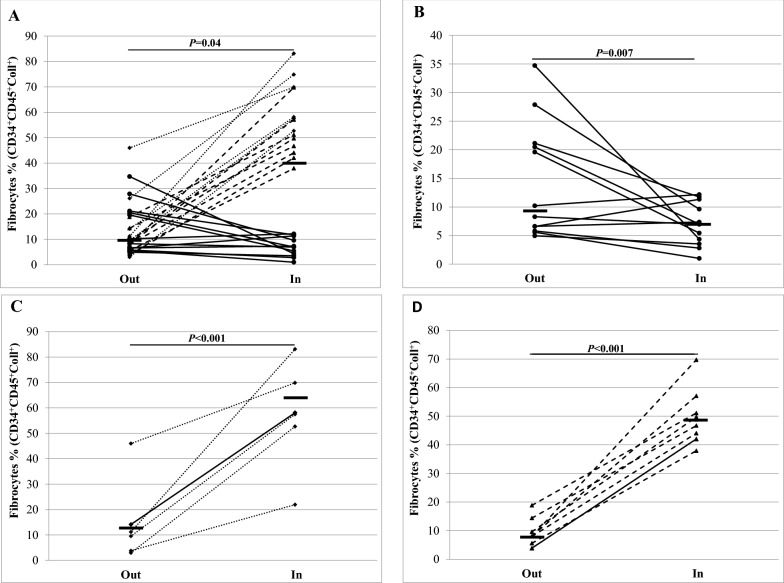
Circulating fibrocytes out vs during pollen season for **A** the whole study population **B** subjects sensitized to tree pollen and being evaluated during the tree pollen season, **C** subjects sensitized to grass pollen and being evaluated during the grass pollen season, and **D** subjects sensitized to ragweed pollen and being evaluated during the ragweed pollen season. The medians are shown by the horizontal lines

The number of circulating fibrocytes significantly increased during the pollen season compared to out of season in the whole study population. When subjects were separated according to the allergen present at the peak of symptoms, the number of fibrocytes significantly decreased for subjects sensitized to tree pollen (April–May) and significantly increased for those sensitized to grass (June–July) and ragweed (August–October) pollen during the pollen season (Figs. 3, 4). The expression of CXCR4 marker on the fibrocytes’ surface significantly decreased during the pollen season in the whole study sample, but according to the allergen type, the decrease was only significant in subjects sensitized to grasses and ragweed (Table 1).

### Mediators’ measurement

There were no significant differences in the concentrations of plasma CXCL12 and of serum MMP9/TIMP1 and SCF between visits (Table 1).

## Discussion

To our knowledge, this study is the first to report changes in circulating fibrocytes during natural allergen exposure in non-asthmatic rhinitic subjects. More interestingly, this variation seems to be different according to the type and duration of seasonal allergen exposure. Hence, two main observations emerge from this study: (1) subjects allergic to trees and who completed the visit at the peak of their symptoms during the tree pollen season showed a decrease in the number of fibrocytes during the pollen season and (2) subjects allergic to grass or ragweed and who completed the visit at the peak of their symptoms during the grass or ragweed pollen season showed an increase in the number of fibrocytes during the pollen season. This suggests a dynamic systemic process potentially contributing to airway remodeling during allergen exposure.

These observations indicate that at the beginning of exposure, there might be an active migration of fibrocytes from the periphery to the bronchial mucosa resulting in a decrease in the number of blood fibrocytes. Following a prolonged pollen exposure, the bone marrow may be more actively stimulated to produce fibrocytes, leading to an increase in the proportion of circulating fibrocytes. Further studies must be conducted to confirm these hypotheses. We cannot exclude a possible “tachyphylaxis” or reduction in bone marrow response in these subjects, with repeated low-dose allergen exposure, as we previously reported with airway eosinophils, but this is unlikely in this setting [22].

Fibrocytes are increased in the bronchial wall according to the severity of asthma [10]. In allergic asthma, allergen exposure can trigger the release of fibrocytes in the blood and their accumulation in the bronchial mucosa [11, 12]. Moreover, a model of allergic asthma showed that fibrocytes are recruited in the bronchial wall post allergen exposure and that they may differentiate into myofibroblasts [9]. The number of myofibroblasts in the airway wall has been found to correlate with the magnitude of sub-epithelial basement membrane fibrosis [8]. Fibrocytes could therefore be described as indirect markers of airway remodeling since they express ECM components and are progenitor cells of myofibroblasts. Hence, they may be seen as interesting surrogates of airway remodeling development in allergic rhinitis.

Thus, our study was based on the hypothesis that the bronchial remodeling process includes, as an initial event, an increase in blood fibrocytes in allergic subjects without asthma, in transit from the bone marrow to the airways. Airway remodeling, on top of which airway inflammation can act, could be one of the factors leading to the increase in airway responsiveness and the eventual development of asthma in some rhinitic subjects. We therefore thought that assessment of blood fibrocytes in rhinitic subjects could provide a non-invasive marker of this process.

We also must acknowledge the limitations of this study. First, we recognize that a laboratory-controlled exposure allows for better control of allergen amounts and duration of exposure, in addition to removing some confounding factors. However, we did not have access to such environmental exposure unit and we believed that the effects of the pollen on circulating fibrocytes were more representative of real-life variations using natural exposure. Second, although our observations show that the allergen exposure has an effect on blood fibrocyte numbers in this population, they cannot confirm if such exposure leads to the migration of circulating fibrocytes into the tissue in response to a remodeling process or even confirm the target site. However, we consider that the bronchial mucosa might be a principal target site since fibrotic changes have already been observed in allergic rhinitis [4], and since our subjects had no other visible wound sites. The nasal mucosa could also be considered as a target site, but the literature is still limited regarding remodeling processes in the upper airways. Thus, even if we still need to determine how these cells are involved in tissue fibrosis in allergic rhinitis, the variation observed in this study supports a role for fibrocytes in its pathophysiology.

Third, 21 of the 26 subjects had perennial allergic rhinitis to either dust or dust mites and/or to cat to which they were exposed at home. This exposure may have influenced the results; however, all subjects were their own control, their pollen season results being compared to their own baseline results. Moreover, all of them had few or no symptoms out of the pollen season and reported more symptoms during the pollen season, as confirmed by the RCSS score.

Fourth, the in-season visit was performed when the subjects reported that their symptoms were at their maximum and when they felt a need to take medication. Even if they were well informed of the main symptoms and if they had certain knowledge of their history of allergic rhinitis, it may have been more accurate to record their symptoms with a more objective method such as daily Total Nasal Symptom Score (TNSS) or daily NPIF. Still, we used the RCSS tool which showed an increase of at least one of the symptoms during the in-season visit, but daily or weekly measures may have been more relevant to demonstrate that subjects had reached the peak of symptoms. Fifth, the worsening of symptoms, shown by the RCSS, is not reflected by a decrease in NPIF at the in season visit. This may be because the NPIF was not performed adequately in this study. In addition to being conducted only once during the pollen season, we believe that some subjects may have blown their nose shortly before the test.

Fibrocytes express CXCR4, the chemokine receptor for CXCL12 which is secreted by endothelial, inflammatory and epithelial cells in the bronchial mucosa of asthmatic patients [23]. Circulating fibrocytes migrate in response to CXCL12 during inflammatory processes and traffic to the lung [24]. CXCR4/CXCL12 seems to be the more specific receptor/ligand axis in the chemotaxis of fibrocytes in asthmatic-exacerbated patients [23]. Our flow cytometry results showed that the number of these receptors decreases when subjects were exposed to grass and ragweed while no change was observed in subjects exposed to tree pollen. Thus, other chemokine axes may be involved, such as the CCL19/CCR7 axis [23]. Moreover, the maturation status of blood fibrocytes may vary according to time and degree of stimulation.

In the present study, the serum concentrations of fibrocytes’ recruitment (SCF) and remodeling (MMP9/TIMP1) markers were not significantly different between visits. SCF may mediate a fibroblasts’ activation pathway in chronic allergic response [25] whereas the role of MMPs and TIMPs, implicated in ECM turnover, is still not completely understood in allergic rhinitis [1]. Even if MMPs are overexpressed in asthmatic subjects [26], they are not upregulated in the nasal mucosa of subjects with perennial allergic rhinitis, and there is no change in the expression of TIMPs [27]. Our results suggest that, in allergic rhinitic subjects, there are no active remodeling processes or that they are not sufficiently intense for these markers to be measurable at least in the serum. In addition, these markers may not be specific enough for these processes to be assessed in the blood. Measurements in the mucosal sites should be more informative.

In conclusion, to our knowledge, this study is the first to evaluate allergen-induced blood fibrocytes changes in a population of allergic rhinitic subjects without asthma during natural allergen exposure. These changes in the number of circulating fibrocytes after allergen exposure could lead to an indirect remodeling process, as it has been demonstrated in asthmatics [8]. However, further studies should be conducted to better understand the role of fibrocytes in allergic rhinitis and their value as markers of airway remodeling. The risk of developing asthma is important for patients with allergic diseases, but we still do not completely know why some patients will do and others will not. By showing that natural allergen exposure leads to a variation in the number of circulating fibrocytes in non asthmatic subjects with allergic rhinitis, these results bring new insight on one of the potential factors involved in asthma development in individuals suffering from seasonal allergic rhinitis.

## Data Availability

The datasets used and/or analysed during the current study are available from the corresponding author on reasonable request.

## Abbreviations

ARIA: Allergic rhinitis and its impact on asthma
ATS: American thoracic society
BSA: Bovine serum albumin
CCL19: C–c-motif chemokine ligand 19
CCR7: C–c-motif chemokine receptor 7
CXCL12: Cxc-motif chemokine ligand 12
CXCR4: Cxc-motif chemokine receptor 4
DTT: Dithiothreitol
ECM: Extracellular matrix
EDTA: Ethylenediaminetetraacetic acid
FBSi: Fetal bovine serum inactivated
FeNO: Fractional exhaled nitric oxide
FEV_1_: Forced expiratory volume in one second
FVC: Forced vital capacity
HBSS: Hanks’ balanced salt solution
IgE: Immunoglobulin E
IUCPQ-UL: Institut Universitaire de Cardiologie et de Pneumologie de Québec—Université Laval
MDD: Minimum detectable dose
MMP9: Matrix metalloproteinase 9
NPIF: Nasal peak inspiratory flow
NS: Not significant
PBMC: Peripheral blood mononuclear cells
PBS: Phosphate buffered saline
PC_20_: Provocative concentration giving a 20% fall in forced expiratory volume in one second
RCSS: Rhinitis control scoring system
SCF: Stem cell factor
SD: Standard deviation
TIMP1: Tissue inhibitor of metalloproteinase 1
TNSS: Total nasal symptoms score
